# Suspected Levonorgestrel-Releasing Intrauterine System (LNG-IUS)-Induced Secondary Adrenal Insufficiency: A Case Report

**DOI:** 10.7759/cureus.66780

**Published:** 2024-08-13

**Authors:** Anthony T Joseph, William Corrigan, Shoaib Asghar, Fernando Abanilla

**Affiliations:** 1 Orthopedic Surgery, Lake Erie College of Osteopathic Medicine, Bradenton, USA; 2 Osteopathic Medicine, Lake Erie College of Osteopathic Medicine, Bradenton, USA; 3 Internal Medicine, Sheikh Zayed Medical College and Hospital, Rahim Yar Khan, PAK; 4 Nephrology, AdventHealth Sebring, Sebring, USA

**Keywords:** levonorgestrel iud, levonorgestrel, dexamethasone supression test, acth, cosyntropin suppression test, secondary adrenal insufficiency, intrauterine contraceptive devices, adrenal insufficiency (ai), central adrenal insufficiency, iud

## Abstract

Central adrenal insufficiency (CAI) is a rare endocrine disorder characterized by insufficient secretion of adrenocorticotropic hormone (ACTH) from the pituitary gland, resulting in decreased cortisol production. Here, we present the case of a 19-year-old female with suspected iatrogenic CAI possibly induced by a levonorgestrel-releasing intrauterine system (LNG-IUS). The patient presented with syncopal events, fatigue, and hypotension, prompting admission to the emergency department. Initial investigations revealed low morning cortisol levels and an inadequate cortisol response to ACTH stimulation, supporting the diagnosis of CAI. Treatment with glucocorticoid replacement therapy led to clinical improvement. Removal of the LNG-IUS led to the recovery of the hypothalamic-pituitary axis. Despite the absence of documented cases of LNG-IUS-induced CAI in literature, similarities with other progestin formulations suggest a potential mechanism involving interaction with glucocorticoid receptors. Further research is needed to elucidate the exact pathophysiology and evaluate the safety of LNG-IUS in relation to the hypothalamic-pituitary-adrenal (HPA) axis. This case underscores the importance of considering hormonal contraceptives as potential triggers of CAI in clinical practice.

## Introduction

Primary adrenal insufficiency is the deficiency in the adrenal gland’s production of mineralocorticoids (aldosterone), glucocorticoids (cortisol), and androgens (testosterone and estrogen), which is often due to autoimmune destruction of the adrenal gland [1]. Secondary adrenal insufficiency or central adrenal insufficiency (CAI) is a subtype of adrenal insufficiency caused by diseases involving the anterior lobe of the pituitary gland, leading to a decreased secretion of adrenocorticotropic hormone (ACTH) [1]. 

Annually, CAI affects roughly 28 per 100,000 individuals, with exogenous cortisol supplementation representing the leading cause of this disorder [2-4]. Unlike primary adrenal insufficiency, which primarily presents in infants, CAI can be asymptomatic or oligo-symptomatic for years before diagnosis [5]. A decreased secretion of ACTH from corticotrophs in the anterior pituitary results in reduced production of glucocorticoids (cortisol) and androgens (testosterone and estrogen). Although many patients are often asymptomatic, non-specific symptoms of CAI may include fatigue, hypotension, hyponatremia, and unintentional weight loss or anorexia [2,6].

While exogenous corticosteroid use remains the most common cause of central adrenal insufficiency, studies have shown that traumatic brain injury (TBI), radiotherapy to the head, and immunotherapy have also been linked to CAI [2]. Newer research has also linked chronic opioid use to the formation of CAI [2,7,8].

For diagnosis of adrenal insufficiency, obtaining basal cortisol levels remains the first step in identifying a hypo-functioning adrenal gland [1]. A cortisol level <3 ug/dL is diagnostic of CAI, while a cortisol level >15 ug/dL allows for exclusion of CAI. Basal cortisol levels between 3-15 ug/dL require further analysis with a 1 ug ACTH stimulation test [2]. After ACTH or cosyntropin stimulation, repeat cortisol at 0 (baseline), 30, and 60 minutes after administration of ACTH, resulting in a cortisol level greater than 18 ug/dL is diagnostic of CAI [9]. Recent evidence has proposed a supraphysiological dose of ACTH as the gold standard for testing CAI [10].

## Case presentation

A 19-year-old female Caucasian patient presented to the emergency department following two syncopal events earlier in the day. Feelings of weakness preceded these syncopal events, but the patient denied any other preceding symptoms and had no confusion following the event. The patient noted that the family witnessed the syncopal episodes, which did not involve any traumatic injury to the head. The patient endorsed a history of fatigue that worsens with exertion, as well as frequent daily naps that lasted for months before admission. The patient also noted reduced strength and weakness with activity. The patient had no significant past medical history besides a levonorgestrel-releasing intrauterine system (LNG-IUS) insertion approximately two years before admission, with an absence of menstrual periods on implantation. The patient's primary care provider referred her to the hospital due to these syncopal episodes and the presence of hypotensive blood pressure readings at the office.

On admission to the emergency department, the patient's vitals showed a blood pressure of 95/48 mmHg, heart rate of 62 bpm, respiratory rate of 19 cpm, and temperature of 98.7 °F. The patient received one liter of normal saline on admission, which provided mild improvement in blood pressure. However, the patient continued to have hypotensive blood pressure readings. Orthostatic vitals were negative following fluid trials. The patient had a normal body mass index (BMI) of 22.3 kg/m³. A physical exam did not reveal any significant findings besides skin flushing. 

A basic laboratory workup was completed on admission to rule out anemia, infection, and electrolyte abnormalities as possible causes of the patient's symptoms. The patient had a normal complete blood count (CBC) workup with no evidence of infectious processes or anemia (Table 1). A basic metabolic panel (BMP) showed no electrolyte abnormalities (Table 2). 

**Table 1:** Complete blood count (CBC) with differential WBC: white blood cells; RBC: red blood cells; MCV: mean corpuscular volume; MCH: mean corpuscular hemoglobin; MCHC: mean corpuscular hemoglobin concentration; RDW: red cell distribution width

Test, Blood	Values	Reference Range
WBC	10.26	3.7 - 10.30 x 10³/uL
RBC	4.40	3.3 - 5.22 x 10⁶/uL
Hemoglobin	13.9	11.2 - 15.7 g/dL
Hematocrit	40.4	34 - 45 %
MCV	91.8	79 - 98 fL
MCH	31.6	26 - 32 pg
MCHC	34.4	30.7 - 35.5 g/dL
RDW	11.9	11.6 - 14.4 %
Platelet Count	323	150 - 400 x 10³/uL
Differential		
Neutrophil %	51.2	34 - 71.1 %
Lymphocyte %	36.5	Female: 19.3 - 51.7 %
Monocyte %	8.7	4.7 - 12.5 %
Eosinophils %	2.6	0.7 - 5.8 %
Basophils %	0.8	0.1 - 1.2 %

**Table 2:** Complete metabolic panel BUN: blood urea nitrogen; AST: aspartate aminotransferase; ALT: alanine transaminase

Test, Serum	Value	Reference Range
Sodium	140	136 - 145 mmol/L
Potassium	4.0	3.5 - 5.0 mmol/L
Chloride	105	98 - 107 mmol/L
Carbon dioxide	24.7	22 - 29 mmol/L
Anion gap	10	5 - 15 mmol/L
BUN	9.2	6 - 20 mmol/L
Creatinine	0.64	0.5 - 1.20 mmol/L
Glucose	101	70 - 100 mg/dL
Calcium	9.8	8.4 - 10.2 mg/dL
Phosphorus	3.62	2.5 - 4.6 mg/dL
Magnesium	2.31	1.70 - 2.80 mg/dL
Albumin	4.78	3.5 - 5.5 g/dL
Protein, total	8.1	6.4 - 8.3 g/dL
AST	16	10 - 42 U/L
ALT	14	10 - 40 U/L

In addition to the CBC and BMP, a urine drug screen (UDS) and beta-human chorionic gonadotropin test (bHCG) were negative. Thyroid-stimulating hormone (TSH) levels were also adequate at 2.35 IU/mL (Table 3).

**Table 3:** Complete endocrine workup ACTH: adrenocorticotrophic hormone; DHEA: dehydroepiandrosterone; LH: luteinizing hormone; FSH: follicle stimulating hormone; GH: growth hormone; TSH: thyroid stimulating hormone; T3: triodothyronine

Hormones	Values	Reference Range
ACTH	2.82	7.2 - 63.3 pg/mL
Cortisol, AM	0.958	6.2 - 19.4 ug/dL
Cortisol, 0 min	3.66	6.2 - 19.4 ug/dL
Cortisol, 30 min	13.82	>18 ug/dL
Cortisol, 60 min	17.43	>18 ug/dL
Aldosterone	9.9	Upright 4.0 - 31 ng/dL
Renin activity	0.7	Upright 0.5 - 4.0 ng/mL/hr
Aldosterone renin ratio	14.14	<25 ratio
17- hydroxyprogesterone	116.93	<206 ng/dL
DHEA sulfate	95	Stage V, female, 65 - 371 ug/dL
LH	3.56	Follicular phase: 2.4 - 12.6 IU/L, Luteal phase: 1.0 - 11.4 IU/L
FSH	3.24	Follicular phase: 3.5 - 12.5 IU/L, Luteal phase: 1.7 - 7.7 IU/L
Progesterone	8.11	Mid-follicular phase: 0.3 - 1.5 ng/mL, Mid-luteal phase: 5.2 - 18.6 ng/mL
GH	0.40	0.01 - 8.00 ng/mL
Somatostatin	23	<30 pg/mL
TSH	2.35	0.34 - 5.6 IU/mL
T3 free	2.72	2.40 - 6.80pg/mL

The patient's ACTH stimulation test indicated an inadequate response of the adrenal glands to a supra-physiological dose of ACTH. With the presence of both inadequate ACTH production by the anterior pituitary and an inadequate response of the adrenal glands to ACTH, a full endocrine panel was ordered to rule out central causes of adrenal insufficiency. However, only progesterone was elevated at 8.11 ng/mL (Table 3).

Following the blood tests and hormone levels, the patient received dexamethasone 4 mg intravenous q6hr. The next day, the patient noted significant improvement in fatigue and weakness they experienced. The blood pressure improved to 105/61 mmHg with a decrease in the number of hypotensive episodes and an improvement in systolic blood pressure. The following day, the patient was switched to oral hydrocortisone for the trial of oral supplementation to transition to discharge. Following the switch to oral steroid supplementation, the patient experienced some episodic hypotension. However, the oral dosage was adjusted, and the patient was discharged home on day four of the hospital stay with the final hydrocortisone dosage of 20 mg (10 mg in AM and up to two doses of 5 mg to be taken as needed for the remainder of the day). Three days after discharge, the patient was referred to her obstetrician for outpatient removal of the intrauterine device (IUD) due to a suspected hormonal negative feedback loop where the levonorgestrel (LNG) was inducing central adrenal insufficiency. During the time between discharge and removal of the IUD, the patient was noted to have persistent lightheadedness. The patient's medication was changed from 20 mg hydrocortisone to 20 mg prednisone (10 mg in the AM followed by 5 mg in the PM and 5 mg before bed). Two weeks later, the patient returned with dizziness, lightheadedness, and weakness despite medical intervention. A urinalysis revealed a UTI causing an adrenal crisis. The patient was educated on the need for increased cortisol when the body is stressed by infection. The patient's medication was changed to 30 mg of hydrocortisone in the AM followed by 15 mg at night, and the patient was given a stress dose of 100 mg of hydrocortisone. The patient was discharged with improvement of symptoms.

Two weeks later, a repeat endocrine panel revealed improving pituitary and adrenal feedback with an AM cortisol level of 8.0 ug/dL, an ACTH level of 6.7 pg/mL, and a negative 21-hydroxylase antibody (Table 4). A normal prolactin level of 15.45 ng/mL was also recorded to complete the endocrine panel (Table 4).

**Table 4:** Adrenal panel ACTH: adrenocorticotrophin hormone

Test	Values	Reference Range
Cortisol, AM	8.0	6.2 - 19.4 ug/dL
ACTH	6.7	7.2 - 63.3 pg/mL
21-hydroxylase antibody	Negative	Negative
Prolactin	15.45	5.15 - 26.52 ng/mL

## Discussion

This case presentation represents a case of suspected iatrogenic CAI believed to be caused by stimulation from the contraceptive LNG-IUS. Although the direct mechanism of LNG-IUS-induced CAI has not been described in the literature to date, other progestin analogs have been implicated in their effects on the HPA axis and involved in CAI through their affinity for glucocorticoid receptors [11,12]. However, current literature on LNG-IUS also provides evidence against the systemic effects of progestin LNG on the hypothalamic-pituitary-adrenal (HPA) axis [13,14]. This discussion aims to explore proposed mechanisms for this CAI case and review the current literature on iatrogenic progestin-induced CAI.

LNG is a second-generation progestin used in different oral, implant, and intrauterine formulations for contraception as well as other pathologies, including endometrial hyperplasia and endometriosis [15]. There are multifocal mechanisms of action proposed for the effectiveness of contraception and reduction of menstrual blood flow achieved by the LNG-IUS. Local endometrial inflammatory reactions, suppressed ovulation, and local hormonal effects on endometrium may all play roles in the LNG-IUS effectiveness as a contraceptive [13]. LNG-IUS has generally been regarded as providing a relatively lower serum concentration of progestins as compared to other contraceptive options, including oral formulations and vaginal release options [15,16]. Because of this, current literature points to the relatively low systemic values of LNG (between 0.3 - 0.5 ug/L) in LNG-IUS patients as evidence against any significant pituitary or systemic effects of progestin [14,15,17]. LNG has agonist activity at both pituitary and nonpituitary progesterone receptors, hence its effectiveness as a contraceptive in systemic (oral) and local release (IUS) formulations. It is also important to note that LNG binds to sex hormone-binding globulin (SHBG) in the systemic circulation, affecting its serum concentration and metabolism [17,18]. 

There is no current case report or literature reporting LNG-IUS-induced CAI. However, current literature suggests CAI is induced by specific oral formulations of the 1^st^ generation progestins, including megestrol acetate (MA) and medroxyprogesterone acetate (MPA) [10,11]. Patients in these cases presented with similar symptoms of adrenal insufficiency, such as palpitations and weakness, as well as laboratory evidence of ACTH and cortisol suppression when compared to this case. An essential aspect of the proposed mechanism of action in these cases was the relatively increased affinity of progestins (46% MA 42% MPA affinity) for glucocorticoid receptors as compared to endogenous cortisol (25% affinity) [10,18]. The proposed mechanism for CAI is as follows: the systemic progestin binding with high relative affinity to glucocorticoid receptors on corticotroph cells of the anterior pituitary, which in turn provides the negative feedback leading to decreased release of ACTH and, in turn, cortisol [11,19,20]. Whether or not this same relationship exists with the relatively low levels of systemic LNG generally seen in LNG-IUS patients is still being determined. Still, more research into the relationship between different progestin formulations, the delivery systems, and the HPA axis is warranted.

## Conclusions

This case report highlights a rare presentation of suspected iatrogenic CAI, possibly induced by LNG-IUS. While the direct mechanism of LNG-IUS-induced CAI remains unclear, the relatively low levels of systemic circulating LNG contradict some aspects of the literature regarding suppression of the HPA axis. LNG, a second-generation progestin, is known for its varied contraceptive applications and local hormonal effects, particularly within the endometrium. Despite generally low systemic concentrations observed with LNG-IUS use, concerns arise from previous reports implicating other progestins in CAI through their interaction with glucocorticoid receptors. This interaction is proposed to suppress ACTH release via negative feedback on corticotroph cells in the pituitary, thereby reducing cortisol production. In this case, the patient had inadequate glucocorticoid production, leading to adverse events, including hypotension and fatigue, with an inability to respond to environmental stressors chemically. The patient's failure to react chemically against environmental stressors was exemplified by the patient's persistent lightheadedness, dizziness, and weakness after developing a UTI despite being on maintenance prednisone dosage. This event provides a scenario where the adrenal gland was insufficient in reacting to an environmental stressor, which required a stress dosage of steroids. While there is no existing literature documenting LNG-IUS-induced CAI, parallels can be drawn with oral progestin formulations that have exhibited similar effects. Differences in progestin types, their delivery systems, and their respective impacts on the HPA axis warrant further investigation to clarify potential mechanisms and risks associated with LNG-IUS use. Continued vigilance and research are essential to enhance our understanding and management of such rare but significant endocrine complications associated with contraceptive practices.
